# Supplementary material for: Universally autonomous self-healing elastomer with high stretchability
Source: Nat Commun. 2020 Apr 27;11:2037. doi: 10.1038/s41467-020-15949-8 (PMC7184568; doi:10.1038/s41467-020-15949-8)
Supplement: Supplementary file 1 — Supplementary Information [file 41467_2020_15949_MOESM1_ESM.pdf]

Supplementary Information for

Universally autonomous self-healing elastomer with high stretchability

Guo *et al.*

Universally autonomous self-healing elastomer with high stretchability

Hongshuang Guo[1, 2, 3, 4], Yi Han [5], Weiqiang Zhao[1, 2, 3, 4], Jing Yang[* 1, 2, 3, 4] and Lei Zhang[* 1, 2, 3, 4]

[1] Department of Biochemical Engineering, School of Chemical Engineering and Technology, Tianjin University, Tianjin 300350, P. R. China

[2] School of Chemical Engineering and Technology, Frontier Science Center for Synthetic Biology and Key Laboratory of Systems Bioengineering (MOE), Tianjin University, Tianjin 300350, P. R. China

[3] Collaborative Innovation Center of Chemical Science and Engineering (Tianjin), Tianjin University, Tianjin, 300350, P. R. China

[4] Qingdao Institute for Marine Technology of Tianjin University, Qingdao 266235, P. R. China.

[5] Tianjin Key Laboratory of Molecular Optoelectronic Science, Department of Chemistry, Tianjin University, Tianjin, 300350, P. R. China

Corresponding Authors: Jing Yang and Lei Zhang

Phone number: 13502038015

*Email address: jing_yang@tju.edu.cn;

lei_zhang@tju.edu.cn.

General Information

Supplementary Methods

Materials. All reagents were commercially available and used as supplied without further purification. 4,4'-bis(methylcarboxy)-2,2'-bipyridine, deuterated solvents were all purchased from Heowns, Tianjin (China). PDMS (M_w = 4600 g mol^{-1}) was purchased from Gelest. IPDI was obtained from Nanjing Sheng Bicheng Chemical Technology Co. Ltd. (China) and dried under vacuum at 80 ℃ before use. DBTDL catalyst was purchased from Adamas. Dichloromethane (CH_2Cl_2), methanol (CH_3OH), hexane, ethanol (CH_3CH_2OH), petroleum ether, sulfuric acid (H_2SO_4), and ethyl acetate were all obtained from Concord Technology. Sodium borohydride ($NaBH_4$), DMAc and SS were purchased from Energy Chemical. BNB was prepared according to published procedures.[1]

Materials Characterization. ^{1}H NMR spectra were recorded on a Bruker Advance 400 MHz spectrometer at room temperature, using the deuterated solvent as the lock and the residual solvent or TMS as the internal reference. GPC tests were carried out using a Shimadzu LC-20AD GPC system. DSC experiments were carried out with a TA Instruments DSC Q2000 system with Tzero aluminum pans. Mechanical tensile-stress experiments were performed using a Shanghai Hualong Electronic Universal Testing Machine. FTIR was conducted on a Bruker Tensor spectrometer (Bruker Optics, Germany) using attenuated total reflectance mode from 4000 to 600 cm^{-1} with a resolution of 4 cm^{-1} and scanning time of 32. The thermal properties of all the polymers were evaluated by thermogravimetric analysis with a differential

thermal analysis instrument (TA Instruments TGA Q50 analyzer) over the temperature range of 20 ℃ – 800 ℃ in an N_2 atmosphere with a heating rate of 10 ℃ min^{-1} (empty Al_2O_3 crucible as the reference). Chemical and elemental analysis of the polymers were examined with XPS (ESCALAB 250 XI, USA) with a Mg *Kα* monochromatic X-ray source. The rheological behavior was recorded on a DHR-2 rheometer. Frequency and temperature sweeps were performed with 2 mm parallel plates on circular samples with a 2 cm diameter. Frequency sweeps at 0.1–100 Hz were measured at 0.1% strain at room temperature (20 ℃). Temperature sweeps were run from 20 ℃ to 80 ℃ at 1 Hz, with the strain automatically modulated at 0.3% ±0.2% by the instrument to keep the measured torque at a reasonable value as the sample softened. Contact with the sample was maintained by the auto-compression feature set to 0.2 ±0.15 N.

Synthetic scheme and NMR spectra

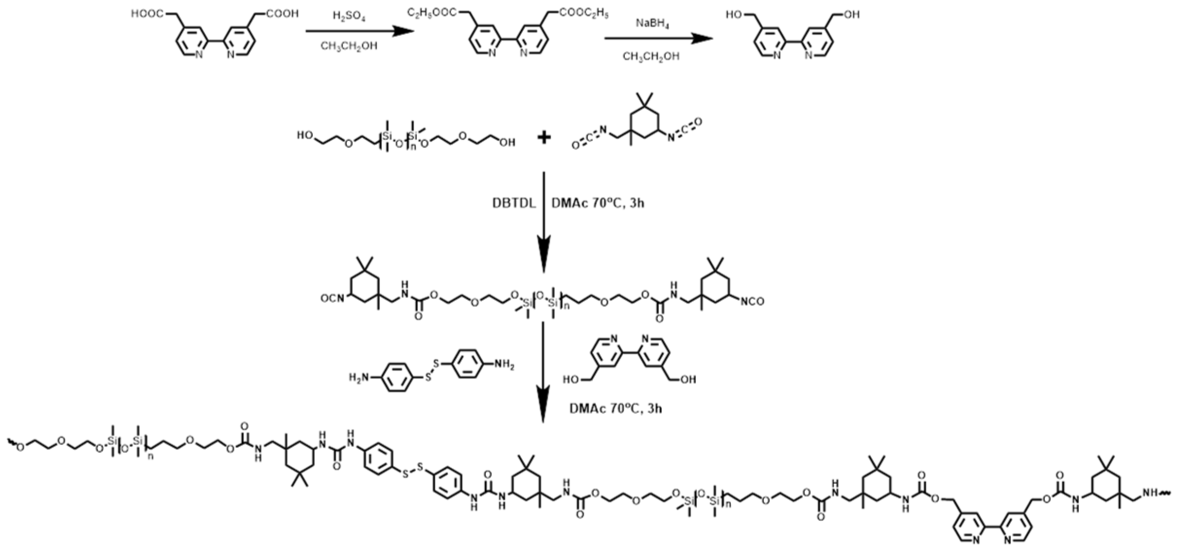

Supplementary Figure 1. Synthetic route of polymeric materials via condensation polymerization.

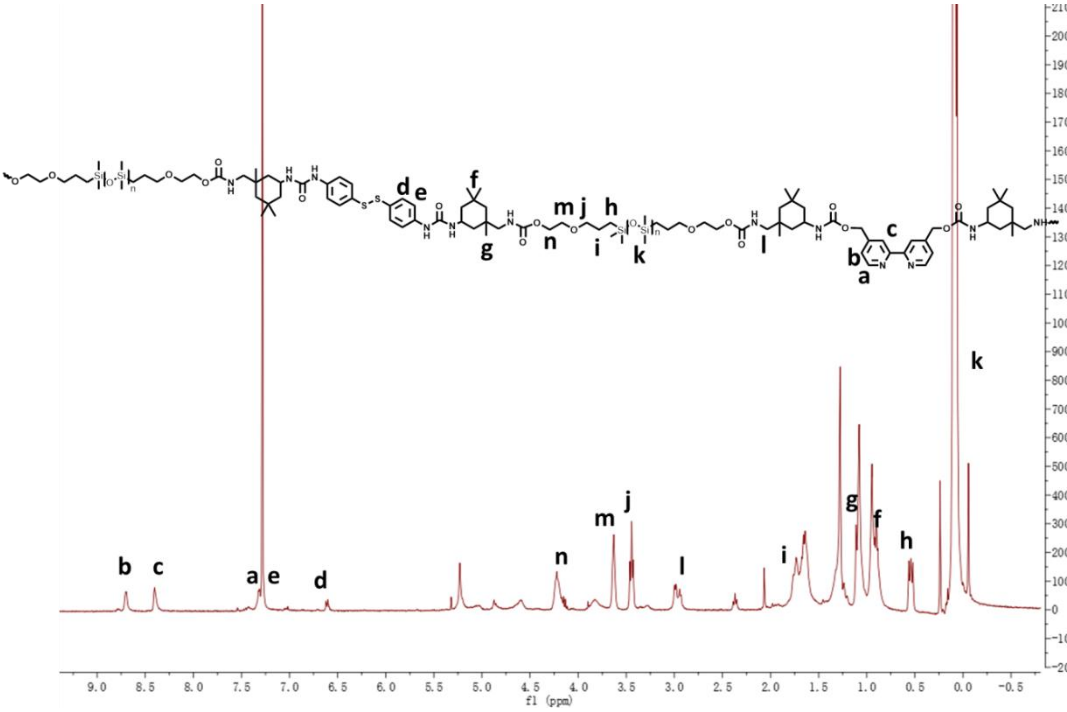

Supplementary Figure 2. ^{1}H **NMR spectrum ($CDCl_3$, room temperature, 400 MHz) of P3.**

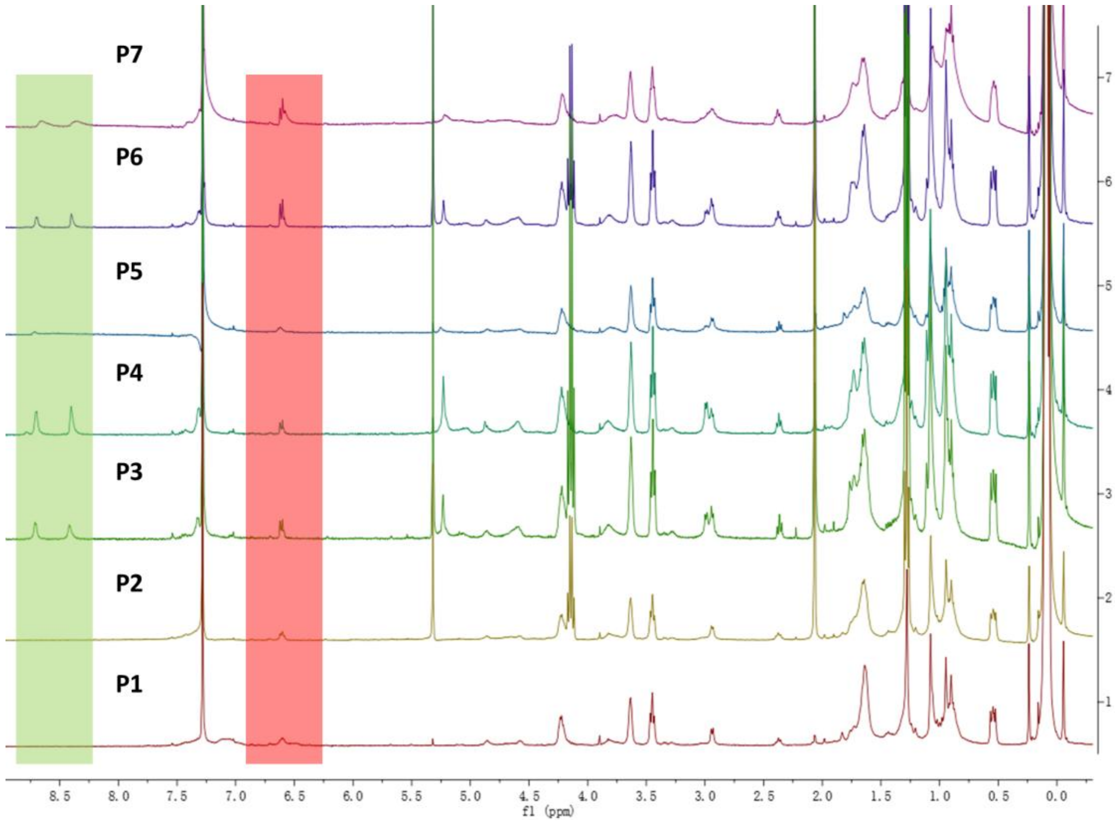

Supplementary Figure 3. ^{1}**H NMR spectra (CDCl$_3$, room temperature, 400 MHz) of P1-7.** The ratios of the signals on 4, 4'-bis(hydroxymethyl)-2,2'-bipyridine (highlighted in green purple) and phenyl ring protons of the 4, 4'-Dithiodianiline unit (highlighted in pink) confirm the incorporation of the corresponding mol% of 4, 4'-bis(hydroxymethyl)-2,2'-bipyridine and 4, 4'-Dithiodianiline into the polymeric backbone.

Supplementary Table 1. The mass ratio of the samples

	PDMS	IP	SS	BNB
P1	1 mmol	2 mmol	1 mmol	0
P2	1 mmol	1.5 mmol	0.5 mmol	0
P3	1 mmol	2 mmol	0.5 mmol	0.5 mmol
P4	1 mmol	2 mmol	0.25 mmol	0.75 mmol
P5	1 mmol	2 mmol	0	1 mmol
P6	1 mmol	4 mmol	2 mmol	1 mmol
P7	1 mmol	2 mmol	0.75 mmol	0.25 mmol

Supplementary Table 2. The mass ratio and molecular weight of the samples

	PDMS:IP:SS:BNB	Mn	Mw	PDI	Young's modulus (Mpa)
P1	1:2:1:0	34002	60746	1.786	0.0105
P3	2:4:1:1	63267	111965	1.769	0.0034
P4	4:8:1:3	66875	111694	1.670	0.0017
P5	1:2:0:1	34624	61840	1.786	0.0081
P6	1:4:2:1	21905	35685	1.629	0.041
P7	4:8:3:1	34038	57512	1.689	0.0075

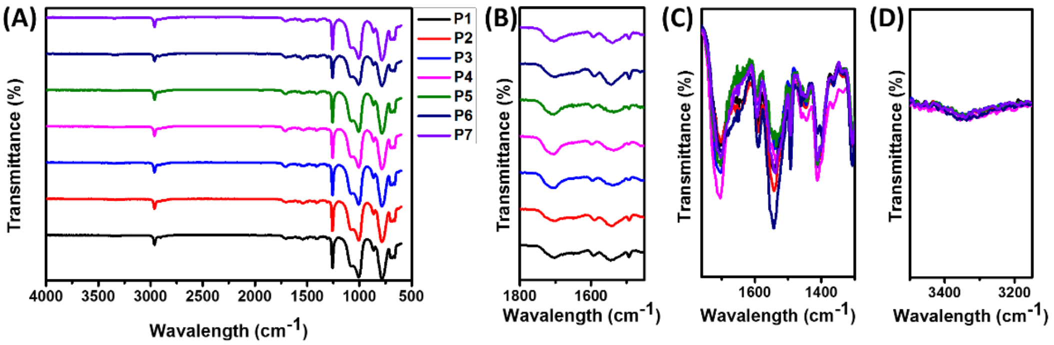

Supplementary Figure 4. FT-IR spectra of P1-7 in the range of 500-4000 cm^{-1}. (A), 1550-1800 cm^{-1} (B) 1300-1760 cm^{-1} (C) and 3100-3500 cm^{-1} (D)**.** Source data are provided as a Source Data file.

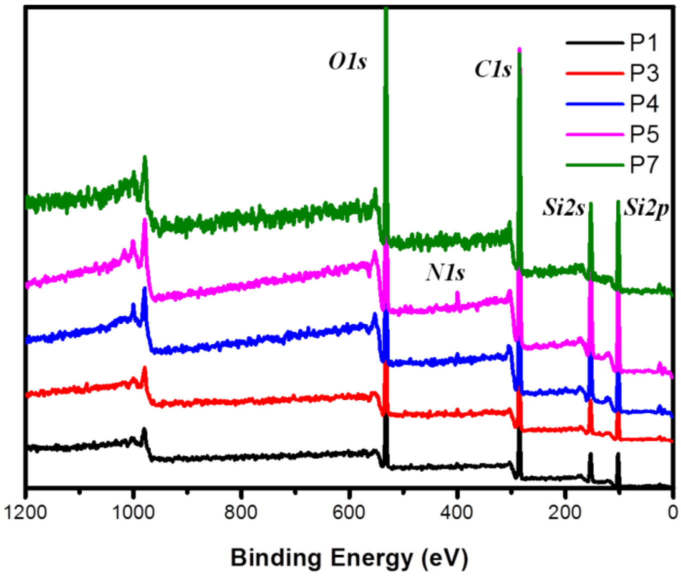

Supplementary Figure 5. XPS graph of P1, P3, P4, P5 and P7. Source data are provided as a Source Data file.

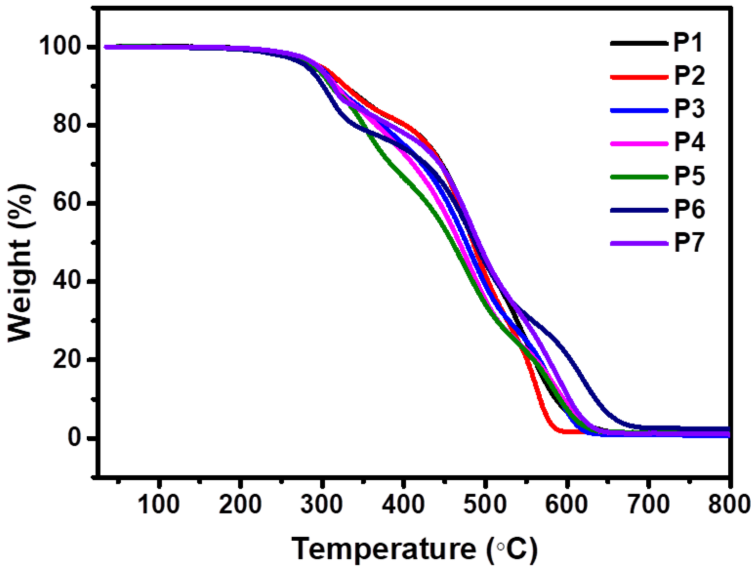

Supplementary Figure 6. TGA graph of P1-7. Source data are provided as a Source Data file.

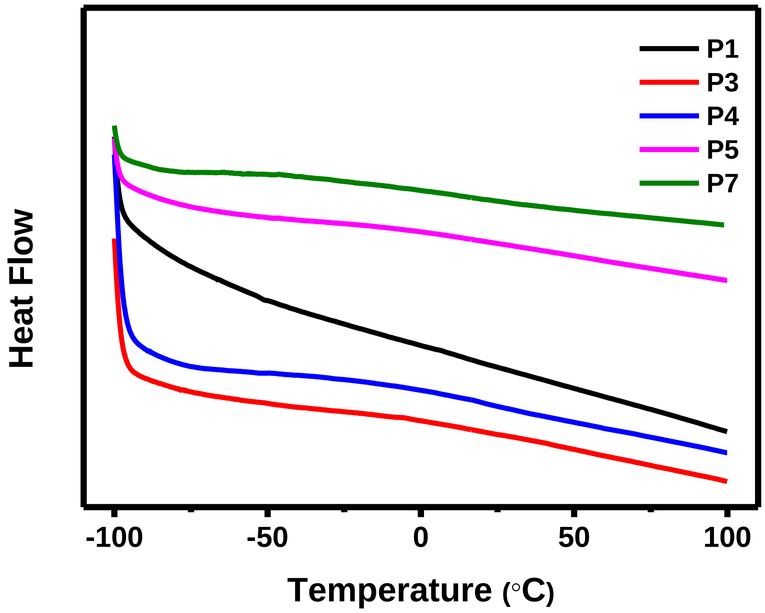

Supplementary Figure 7. DSC heating curves of P1, P3, P4, P5 and P7. After eliminating thermal history, data are collected during the second heating process from -100 to 100 ℃ at a constant rate of 10 ℃ min^{-1}. Source data are provided as a Source Data file.

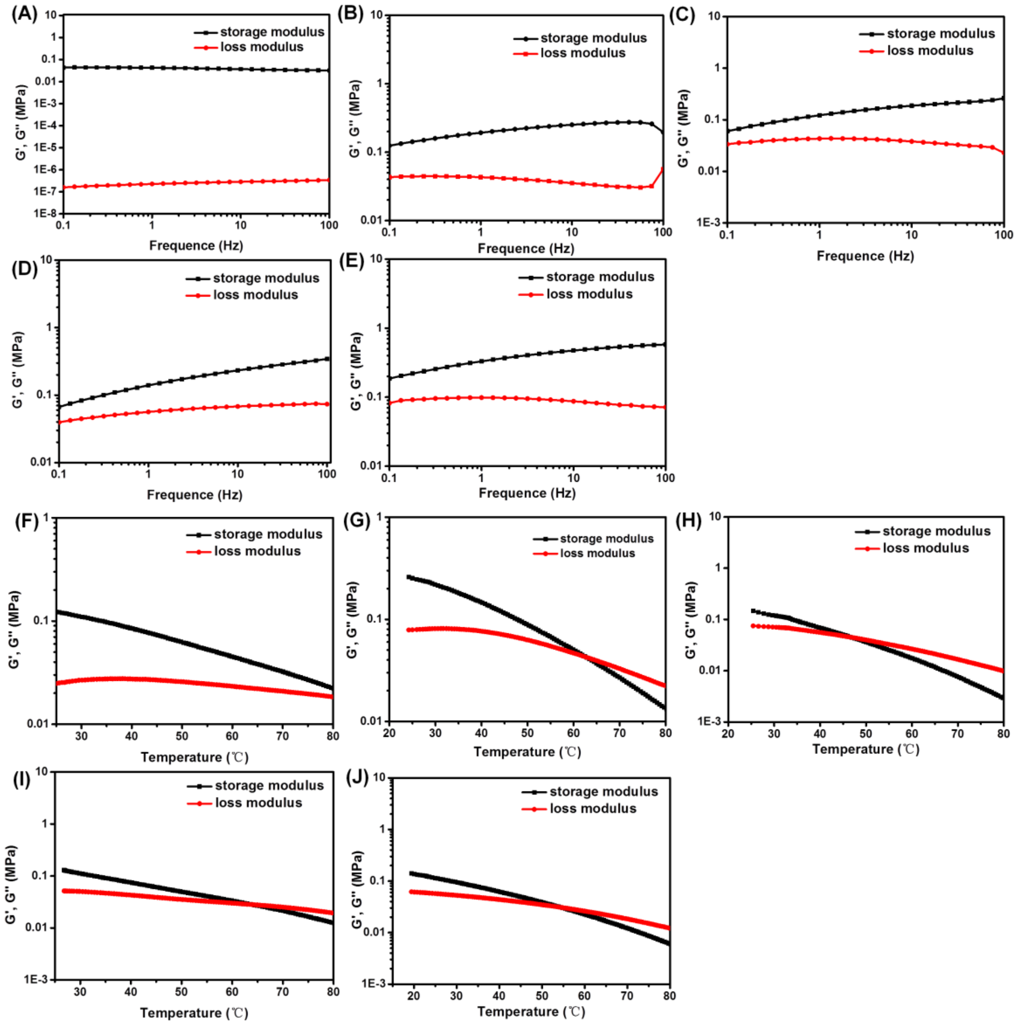

Supplementary Figure 8. The rheological test of P1, P3, P4, P5 and P7 films. A-E). Loss modulus (G") and storage modulus (G') of films versus frequency at room temperature (20 ℃). **F-J).** G" and G' of films versus temperature at 1 Hz. Source data are provided as a Source Data file.

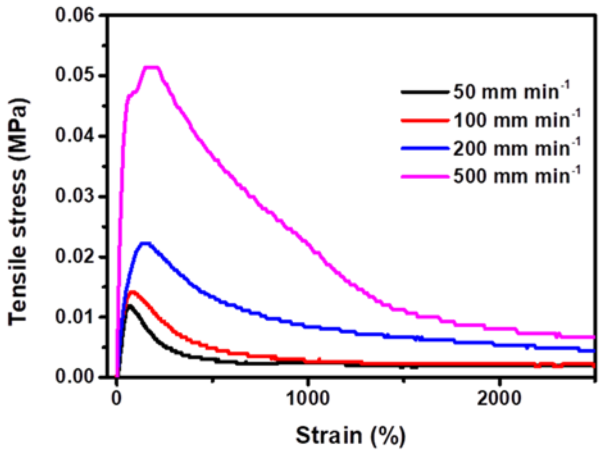

Supplementary Figure 9. Stress-strain curve of the film P4 stretched at different speeds for a sample of width 14 mm, thickness 1 mm and gauge length 2 mm. Source data are provided as a Source Data file.

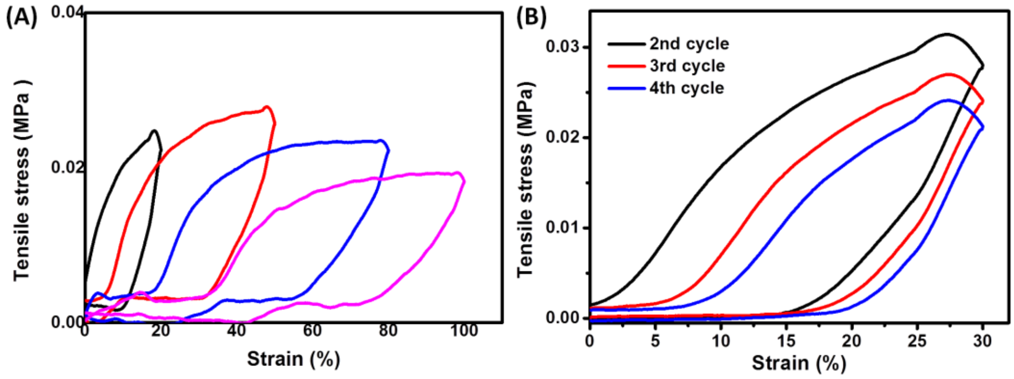

Supplementary Figure 10. (A). The stress-strain curve of the P4 film in stress-strain tests (20%, 50%, 80%, 100% strain) in successive stretching; (B). The stress-strain curve of the P4 film in cyclic stress-strain tests (30% strain) in successive stretching. Sample width: 14 mm; Thickness: 1 mm; Gage length: 40 mm; Stretching speed: 10 mm min^{-1}. Source data are provided as a Source Data file

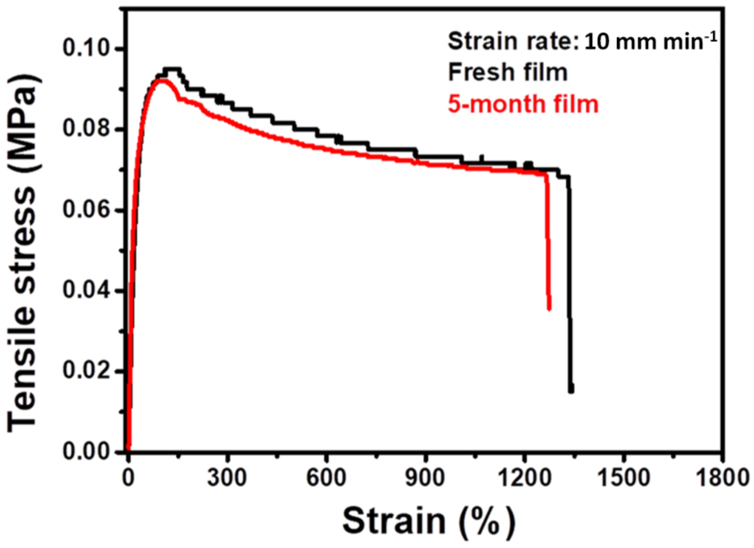

Supplementary Figure 11. Stress-strain curves of the fresh P3 film and one stored for ~5 months. Source data are provided as a Source Data file

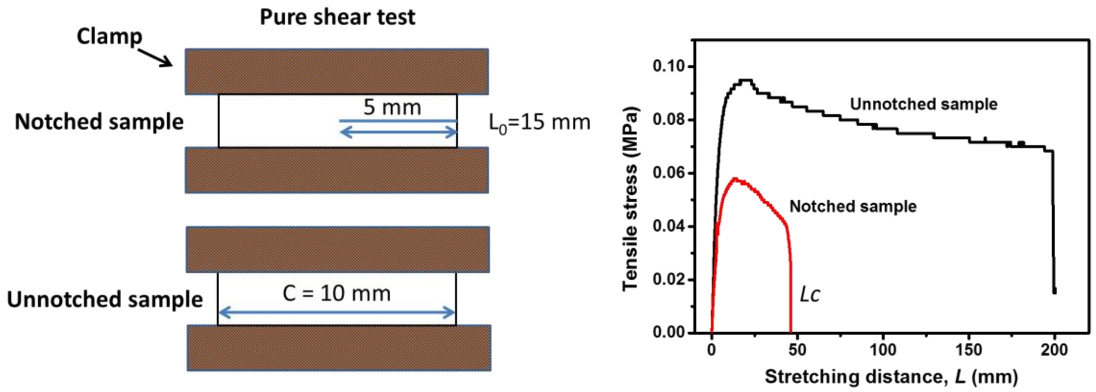

Supplementary Figure 12. Geometry of unnotched and notched samples for pure shear test. Sample thickness, 2 mm. Stress–extension curves of the unnotched and notched P3 film. *Lc* is the distance between the clamps when a crack starts to propagate. The inset graph is the schematic diagram of notch location**.** Source data are provided as a Source Data file.

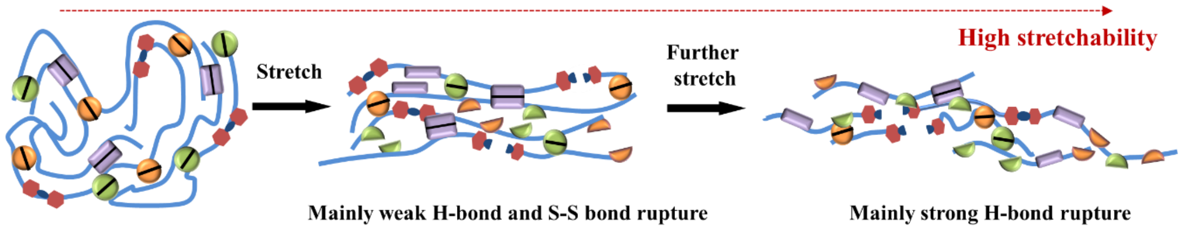

Supplementary Figure 13. A proposed mechanism for chain folding and sliding during tensile stretching.

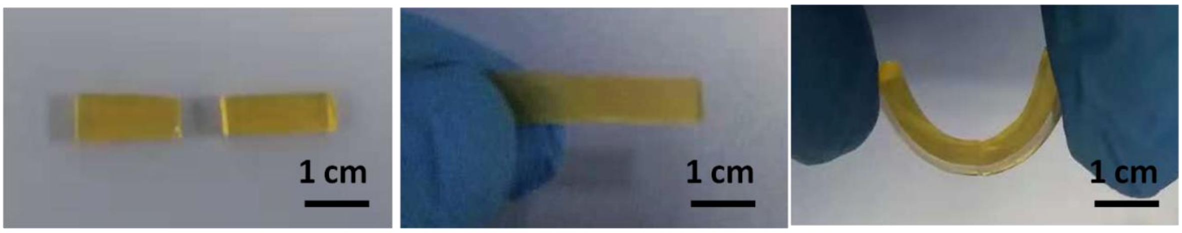

Supplementary Figure 14. Photograph of P3 film before and after self-healing

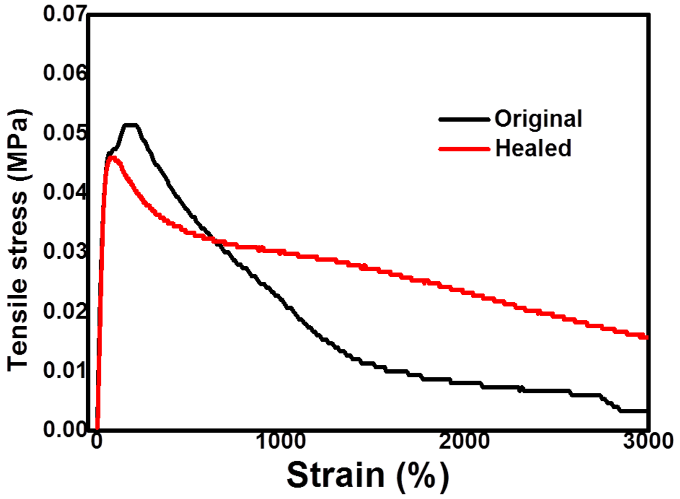

Supplementary Figure 15. The stress-strain curves of P4 film healed at room temperature for 12 h. Sample size: 60 × 20 × 1.0 mm^3; Gage length: 2 mm; Stretching speed: 100 mm min^{-1}. The healing of P4 film is quick and efficient. The film healed at room temperature for 12 hours can be stretched to over 30 times of its original length without breaking. Source data are provided as a Source Data file.

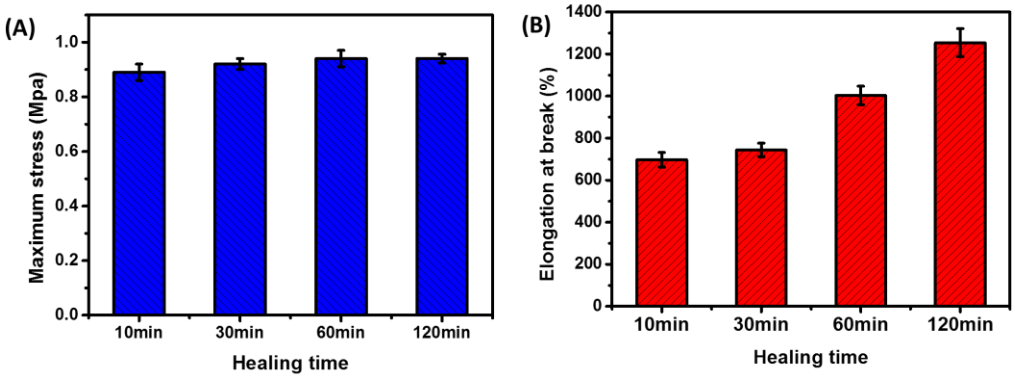

Supplementary Figure 16. Dependence of maximum stress and elongation at break on the healing time of P3 film healed at room temperature (A) maximum stress and (B) elongation at break. Sample size: 60 × 20 × 1.0 mm^3; Stretching speed: 10 mm min^{-1}. Error bars = standard deviation (n = 3). Source data are provided as a Source Data file.

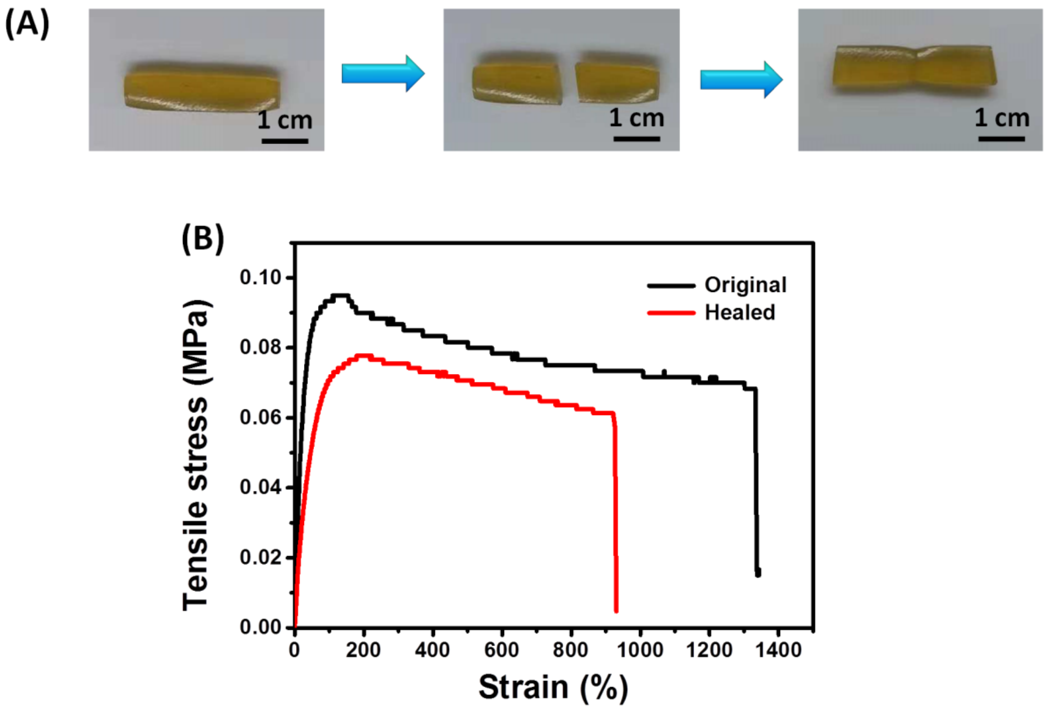

Supplementary Figure 17. A) The photograph of the procedures for self-healing from undamaged surface. B) The stress-strain curves of P3 film healed by undamaged surface at room temperature for 24 h. Sample size: 75 ×14 ×1.0 mm^3; Gage length: 2 mm; Stretching speed: 10 mm min^{-1}. Source data are provided as a Source Data file.

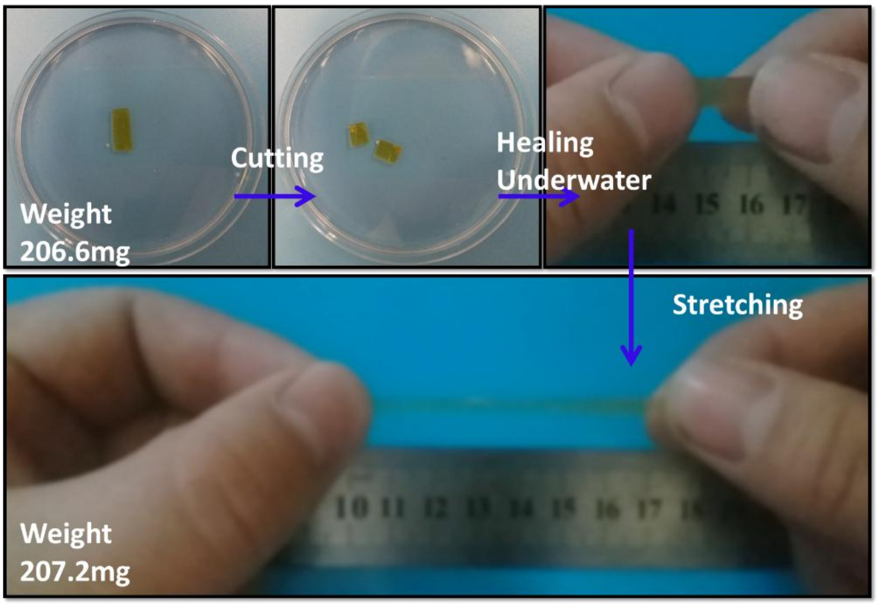

Supplementary Figure 18. Underwater self-healing test. Original P3 film is bisected to two pieces and put together underwater for self-healing. After 24 hours, film is successfully stretched.

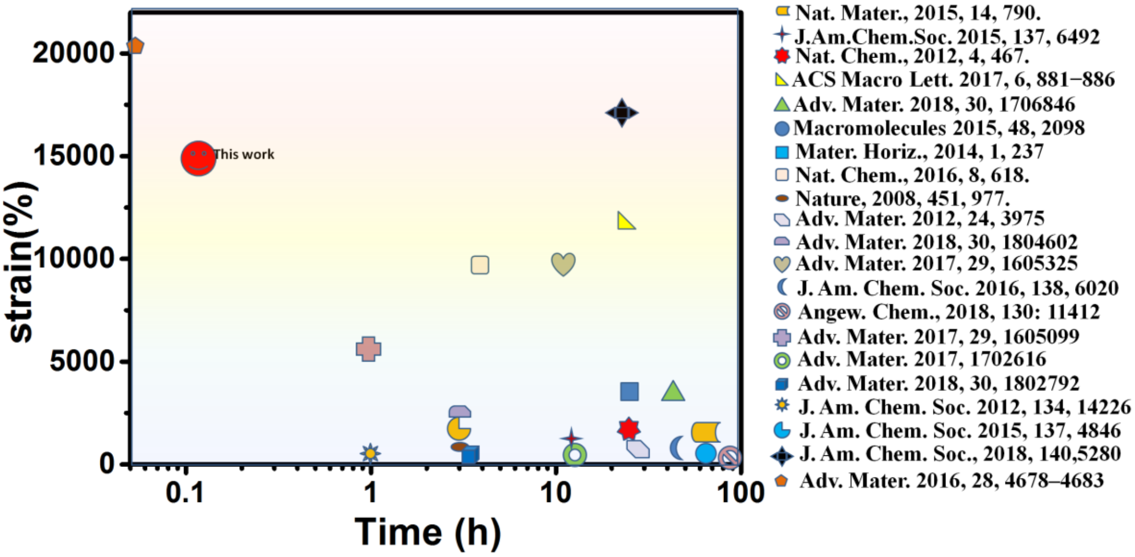

Supplementary Figure 19. Graphic comparison of the stretchability and self-healing time of the PDMS–SS–IP–BNB elastomer and many classical stretchable elastomers and tough hydrogels reported in the literature (ref.[2-22]).

Supplementary References:

1 Miyoshi, D., Karimata, H., Wang, Z.-M., Koumoto, K. & Sugimoto, N. Artificial G-Wire Switch with 2,2'-Bipyridine Units Responsive to Divalent Metal Ions. *J. Am. Chem. Soc.* **129**, 5919-5925, (2007).

2 Yan, X. et al. Quadruple H-Bonding Cross-Linked Supramolecular Polymeric Materials as Substrates for Stretchable, Antitearing, and Self-Healable Thin Film Electrodes. *J. Am. Chem. Soc*. **140**, 5280-5289, (2018).

3 Rao, Y.-L. et al. Stretchable Self-Healing Polymeric Dielectrics Cross-Linked Through Metal–Ligand Coordination. *J. Am. Chem. Soc*. **138**, 6020-6027, (2016).

4 Cordier, P., Tournilhac, F., Soulié-Ziakovic, C. & Leibler, L. Self-healing and thermoreversible rubber from supramolecular assembly. *Nature* **451**, 977, (2008).

5 Chen, Y., Kushner, A. M., Williams, G. A. & Guan, Z. Multiphase design of autonomic self-healing thermoplastic elastomers. *Nat. Chem*. **4**, 467, (2012).

6 Rekondo, A. et al. Catalyst-free room-temperature self-healing elastomers based on aromatic disulfide metathesis. *Mater. Horiz*. **1**, 237-240, (2014).

7 Li, C.-H. et al. A highly stretchable autonomous self-healing elastomer. *Nat. Chem*. **8**, 618, (2016).

8 Neal, J. A., Mozhdehi, D. & Guan, Z. Enhancing Mechanical Performance of a Covalent Self-Healing Material by Sacrificial Noncovalent Bonds. *J. Am. Chem. Soc*. **137**, 4846-4850, (2015).

9 Cui, J., Daniel, D., Grinthal, A., Lin, K. & Aizenberg, J. Dynamic polymer systems with self-regulated secretion for the control of surface properties and material healing. *Nat. Mater.* **14**, 790, (2015).

10 Cromwell, O. R., Chung, J. & Guan, Z. Malleable and Self-Healing Covalent Polymer Networks through Tunable Dynamic Boronic Ester Bonds. *J. Am. Chem. Soc.* **137**, 6492-6495, (2015).

11 Wang, P., Deng, G., Zhou, L., Li, Z. & Chen, Y. Ultrastretchable, Self-Healable Hydrogels Based on Dynamic Covalent Bonding and Triblock Copolymer Micellization. *ACS Macro Letters* **6**, 881-886, (2017).

12 Kang, J. et al. Tough and Water-Insensitive Self-Healing Elastomer for Robust Electronic Skin. *Adv. Mater.* **30**, 1706846, (2018).

13 Cash, J. J., Kubo, T., Bapat, A. P. & Sumerlin, B. S. Room-Temperature Self-Healing Polymers Based on Dynamic-Covalent Boronic Esters. *Macromolecules* **48**, 2098-2106, (2015).

14 Amamoto, Y., Otsuka, H., Takahara, A. & Matyjaszewski, K. Self-Healing of Covalently Cross-Linked Polymers by Reshuffling Thiuram Disulfide Moieties in Air under Visible Light. *Adv. Mater.* **24**, 3975-3980, (2012).

15 Cao, Y. et al. A Highly Stretchy, Transparent Elastomer with the Capability to Automatically Self-Heal Underwater. *Adv. Mater.* **30**, 1804602, (2018).

16 Liu, J. et al. Tough Supramolecular Polymer Networks with Extreme Stretchability and Fast Room-Temperature Self-Healing. *Adv. Mater.* **29**, 1605325, (2017).

17 Liu, M., Liu, P., Lu, G., Xu, Z. & Yao, X. Multiphase-Assembly of Siloxane Oligomers with Improved Mechanical Strength and Water-Enhanced Healing. *Angew. Chem. Int. Ed*. **130**, 11412-11416, (2018).

18 Cao, Y. et al. A Transparent, Self-Healing, Highly Stretchable Ionic Conductor. *Adv. Mater*. **29**, 1605099, (2017).

19 Wu, J., Cai, L.-H. & Weitz, D. A. Tough Self-Healing Elastomers by Molecular Enforced Integration of Covalent and Reversible Networks. *Adv. Mater*. **29**, 1702616, (2017).

20 Tamate, R. et al. Self-Healing Micellar Ion Gels Based on Multiple Hydrogen Bonding. *Adv. Mater*. **30**, 1802792, (2018).

21 Lu, Y.-X. & Guan, Z. Olefin Metathesis for Effective Polymer Healing via Dynamic Exchange of Strong Carbon–Carbon Double Bonds. *J. Am. Chem. Soc*. **134**, 14226-14231, (2012).

22 Jeon, I., Cui, J., Illeperuma, W. R. K., Aizenberg, J. & Vlassak, J. J. Extremely Stretchable and Fast Self-Healing Hydrogels. *Adv. Mater*. **28**, 4678-4683, (2016).
